# The Dark Side of Mobile Learning via Social Media: How Bad Can It Get?

**DOI:** 10.1007/s10796-021-10202-z

**Published:** 2021-10-09

**Authors:** Xiu-Kin Loh, Voon-Hsien Lee, Xiu-Ming Loh, Garry Wei-Han Tan, Keng-Boon Ooi, Yogesh K. Dwivedi

**Affiliations:** 1 Faculty of Business and Finance, Universiti Tunku Abdul Rahman, Jalan Universiti, 31900 Bandar BaratKampar, Perak Malaysia; 2 UCSI Graduate Business School, UCSI University, No. 1 Jalan Menara Gading, UCSI Heights, 56000 Cheras, Wilayah Persekutuan Kuala Lumpur Malaysia; 3 School of Finance and Economics, Nanchang Institute of Technology, 901 Ying Xiong Avenue, Chang Bei Economic Development Zone, Nan Chang City, 330034 Jiang Xi Province People’s Republic of China; 4 College of Management, Chang Jung Christian University, Guiren District, Tainan City, 711 Taiwan Taiwan; 5 School of Management, Emerging Markets Research Centre (EMaRC), Swansea University Bay Campus, Fabian Way, Swansea, SA1 8EN UK; 6 Department of Management, Symbiosis Institute of Business Management, Pune & Symbiosis International (Deemed University), Pune, India

**Keywords:** Stimulus–organism–response, Technostress, Exhaustion, Higher education, Online learning, Mobile learning, Distance learning, Social media, COVID-19

## Abstract

As the COVID-19 pandemic continues to spread at an unprecedented rate, many universities around the world halted physical forms of teaching and learning to stop the spread of the virus. As a result, many university students were forced to utilize online learning through channels such as mobile social media. Due to the novelty of this situation, there are many unknowns particularly with the negative influences of mobile learning via social media on university students. Thus, this study looks to examine this subject matter from the perspective of the stimulus–organism–response theory. The uniquely developed research model included four stimuli (i.e., social overload, information overload, life invasion, and privacy invasion), two organisms (i.e., technostress and exhaustion) as well as a response in terms of reduced intention to use mobile learning via social media. The responses were collected from 384 university students via an online survey and analyzed with the Partial-Least-Square-Structural-Equation-Modelling. It was found that the antecedents for both technostress and exhaustion were able to account for more than half of their respective variances. Furthermore, technostress and exhaustion were significant facilitators of the students’ reduced intention to use mobile learning via social media. In addition to the practical insights for stakeholders in the education industry, this study also posited several theoretical implications for researchers.

## Introduction

Since its emergence, social media has now become a mainstay for many people around the world. Statistics reveal that 55% of the world’s population are active social media users (Kemp, 2021a). Particularly in Malaysia, the percentage of Malaysians who are active social media users stands at 86%. In addition, when it comes to accessing social media, 99% of active users in Malaysia do so via their mobile phones (Kemp, 2021b). The prevalence of social media is credited to its effectiveness in proliferating into virtually every aspect of our everyday lives via mobile devices (Balakrishnan & Gan, 2016; Dwivedi et al., 2021). Nowadays, many individuals use social media for educational purposes. This is referred to as online learning which brings about many benefits such as convenience, flexibility, and so on (Giannakos et al., 2021).

Online learning (e-learning) has recently taken center stage in higher education due to the COVID-19 pandemic (Barnes, 2020; Davison, 2020; Dwivedi et al., 2020; Krishnamurthy, 2020; Mittal et al., 2021). This is because many universities around the world had to close their campuses and cease on-site activities in an effort to stop the spread of the virus (Banoo, 2020). In Malaysia, the effort to stop the spread of the virus was through the enactment of the Movement Control Order and its variants. This required all universities in the country to conduct teaching and learning activities through online channels. In addition, this situation is expected to continue for a long period of time in accordance with the instructions by the Ministry of Higher Education in Malaysia (Palansamy, 2020). As a result, students have no other choice but to use online platforms for learning (Lee & Trimi, 2020). Given the high smartphone penetration (99.2%) and social media users (86.0%) in Malaysia (Kemp, 2021b), this has been supplemented by mobile social media as a tool for pedagogy (Kapoor et al., 2018).

However, given the abruptness and novelty of the situation, the utilization of mobile learning via social media would result in more negative effects instead of positive ones to the students (Lim, 2020). For example, the ubiquitous and social nature of mobile social media enables students to contact each other at any time and view their friends’ posts on the same platform that they are using to study. Hence, there is a greater integration of the social element into the learning process which may serve as a distraction to the students (Gupta & Irwin, 2016). Moreover, some instructors provide a lot of supplementary materials for their students to learn on social media and carry out classes during weekends and public holidays. Therefore, the objective of this study is to determine the variables that affect university students’ reduced intention to use mobile learning via social media in Malaysia during a pandemic.

Furthermore, studies on the reduced intention to use mobile learning via social media are scarce. This is further compounded when narrowing down the scope to studies that were conducted in Malaysia which utilized the SOR theory in the midst of a pandemic. Therefore, this study looks into the causes of the negative impacts and how they reduce university students’ intention to use mobile learning via social media. The insights from this study will be of great value to many stakeholders especially educators in riding out the storm caused by the COVID-19 pandemic. In particular, university instructors in other countries that utilize mobile social media for teaching purposes due to the lack of a proper online education management system can also benefit from the insights of this study. This study serves to greatly extend the comprehension of factors that would reduce students’ intention to use mobile learning via social media given the scarcity of studies on the subject matter. More specifically, the conceptual model of this study highlights the antecedents and influences of technostress (TS) and exhaustion (EXH) on the university students’ intention to use mobile learning via social media.

## Literature Review

### Mobile Learning

Mobile learning is a subcategory of e-learning (Basak et al., 2018) that refers to the learning of knowledge and skills with a mobile device (e.g., smartphone, tablet) (Yeap et al., 2016). However, mobile learning is significantly unique given the technical portability and ubiquity of smartphones and tablets (Sharples et al., 2005). It enables students to transcend time and space when it comes to learning. In particular, they have the opportunity to learn beyond the confines of a physical classroom and at any time of the day (Sharples et al., 2009). Kumar and Chand (2018) in their systematic literature review found an increase in research on mobile learning but the studies were mainly focused on adoption and tend to utilize facilitating factors. Since then, there were a number of studies published in the area of mobile learning such as Al-Azaweia and Alowayr (2020), Thongsri et al. (2018), as well as Almaiah et al. (2019). Particularly when it comes to social media in the academic context, there are several prevalently utilized platforms such as Facebook, YouTube, WhatsApp, and others that enable instructors to make important announcements, carry out online discussions, and disseminate resources (Wang et al., 2012). Thus, mobile learning and SNS can be considered to be effective educational tools that play crucial roles in the modern education setting (Basak et al., 2018). In Malaysia, Moorthy et al. (2019) and Moghavvemi et al. (2017) looked into the intention to use social media for learning in the context of tertiary education. However, these studies are generally similar to the trends identified in the above-mentioned systematic review of literature in view of their focus on adoption and facilitating factors.

Given the above, the inclusion of mainly facilitating variables in the research models of the aforesaid past studies is posited as a detriment. This is because of the pro-change bias that occurs when it is assumed that individuals are generally open to change and would lean towards adopting or continuing their use of innovative technology. In reality, it tends to be the opposite as it is a natural response for individuals to resist change (Talke & Heidenreich, 2014). As such, it is important to examine the factors that can cause users to reduce their use of social networking sites (SNS). In addition, the emphasis on adoption is opined as irrelevant in the setting of this study as students are required to use mobile learning due to the COVID-19 pandemic. Given that students are already using mobile learning, this study looks into the “acceptance-discontinuance anomaly”. This occurs when the students develop an intention to reduce or discontinue their use of mobile learning after the initial acceptance (Bhattacherjee, 2001). Specifically in the context of this study, the initial acceptance was forced upon by the pandemic. Overall, this study serves to fill the literature gaps by addressing the pro-change bias present in past studies and identify the reasons behind why students would reduce their use of mobile learning.

### Stimulus–Organism–Response (SOR) Framework

The SOR framework was developed by Mehrabian and Russell (1974) to study the domain of environmental psychology. This framework posits that cues (stimulus) from the environment can affect changes in a person’s internal state (organism). This will result in a positive or negative behavioral outcome (response) (Mehrabian & Russell, 1974). More specifically, a stimulus is an influencing variable of the external environment that can exert influence on the person’s cognitive, mental, and emotional state (Liu et al., 2018). This will then be manifested as an active response to external stimuli in terms of the person’s specific behavior (Kamboj et al., 2018; Zhu et al., 2020).

Since its conception, this framework has been recently used to analyze user behaviors in the e-learning setting. For example, the SOR framework has been utilized by Zhai et al. (2020) to examine e-learning behaviors and outcomes. One of the study’s key results is the vital significance that privacy concern has with the subject matter. Other recent studies include Zhao et al. (2020) on the continuance intention to use massive open online courses as well as Yang et al. (2019) on the continuance intention to use mobile learning.

The SOR framework is utilized in this study as mobile learning via social media is an external environment of students that alludes to stimuli in association with its use. These stimuli will then have corresponding impacts on the students’ state which would ultimately lead to their response. Particularly in this study, four constructs serve as stimuli which are social overload (SO), information overload (IO), life invasion (LI), and privacy invasion (PI). SO describes the negative effects of crowding in online platforms where “users perceive too many social demands to process and perceive that they have to invest too much time and attention to maintain relationships with their growing number of contacts in the online social network” (Zhang et al., 2016, p. 6). Moreover, IO refers to the feeling of being overwhelmed because of the ever-increasing amount of information present over the Internet (Ragu-Nathan et al., 2008). Besides, LI refers to the invasive nature of information technology that blurs the boundaries between personal and work life as users become reachable anytime and anywhere (Xiao & Mou, 2019). Besides that, PI refers to the concern that user’s personal information such as personal profiles, private messages, uploaded contents may be leaked to the public (Kim et al., 2019; Zhou & Li, 2014).

For organisms, technostress (TS) and exhaustion (EXH) are postulated as the internal states experienced by students when using mobile learning via social media. While TS simply means a state of stress experienced by users psychologically when they engage in technology use (Cao & Sun, 2018), EXH is indicated by their “feelings of being exhausted and bored while using social networking sites” (Luqman et al., 2017, p. 546). Subsequently, the response is externalized as the reduced intention to use (RIU) mobile learning via social media. In this study, the emphasis is on avoidance behavior which is known as a form of disengagement coping. More precisely, avoidance behavior is characterized as an individual’s attempt to reduce the experience of undesirable emotions by distancing or totally removing themselves from that stimulus (Hofmann & Hay, 2018). In other words, individuals will avoid situations that are exhausting and stressful by committing to behavioral change. Those who experience negative emotions will consider minimizing the influences of these emotions by controlling their usage (Yao & Cao, 2017). For them, this serves as a strategy to decrease or eliminate the negative repercussions brought about by technology as well as to reinforce their emotional steadiness (Cao & Sun, 2018).

### Reduced Intention to Use (RIU)

There are two major strategies that users can utilize when dealing with technological-related threats, namely disturbance handling and self-preservation (Beaudry & Pinsonneault, 2005). In the context of this study, as university students have very limited control over the entire situation, they would have to employ the self-preservation strategy. This strategy is an emotion-focused adaptation in which the users focus on regaining their emotional stability by minimizing the emotional adversity associated with the use of a particular technology (Chen et al., 2019). It is also known as disengagement coping and can be externalized in a number of avoidance behaviors such as reducing the use, taking a short break, and suspending the use (Osatuyi & Turel, 2020; Ravindran et al., 2014). From the above-mentioned, this research looks to capture the desire of university students to partially or totally reduce their use of mobile learning via social media through the conceptualization of RIU. Thus, the application of RIU in this study refers to the university students’ intention to decrease their usage intensity or stop their use of mobile learning via social media.

## Hypotheses Development

### Stimulus–Organism Relationship

In the context of mobile learning via social media, social overload (SO) refers to the situation where a person feels overwhelmed by the large amount of social demand entrusted to him or her (Shi et al., 2020). Students engaging in mobile learning via social media would experience SO as they participate in collaborative learning (Sarwar et al., 2019). This would involve regular communication among students via chat and online communities on social media platforms (Fu et al., 2020). With the increased social interactions, students encounter the pressures of social norms to respond to each other’s social demands. This is because for them to maintain relationships, they have to fulfil as many of these social demands as they can. Furthermore, given the nature of mobile devices, these social demands can arise anytime and anywhere (Shi et al., 2020). Therefore, they habitually check their social media so that they can avoid missing out on any updates and respond to messages immediately to stay in contact with friends. Hence, frequent notifications from social media can result in cognitive burdens among students (Cao & Sun, 2018). Ultimately, the negative stimulus of SO will lead students to experience negative states of TS and EXH (Maier et al., 2015; Zhang et al., 2016). Hence, the hypotheses below were developed:

#### H1a:

Social overload has a significantly positive relationship with technostress.

#### H1b:

Social overload has a significantly positive relationship with exhaustion.

Information overload (IO) is defined as the level to which the amount of information is beyond the person’s ability to manage it (Guo et al., 2020). Given the nature of social media and its prevalence in the lives of students, they receive information at an exceptional rate (Shi et al., 2020). This is because both students and educators can generate educational information and share the content online such as study materials, announcements from the instructors as well as instructions on the subject, course, assignment, and so on (Dany, 2019). Consequently, mobile social media applications will notify students of such information. As students have a habit of checking their notifications regularly, they would receive and engage with more information than they would prefer (Salo et al., 2019). However, students only have limited capabilities to deal with the influx of information (Fu et al., 2020). Hence, the constantly increasing levels of information disseminated through social media can quickly overwhelm the students’ cognitive threshold. Thus, the overwhelming influx of information will lead to the negative states of TS and EXH (Cao & Sun, 2018; Shi et al., 2020). As such, the hypotheses below were developed:

#### H2a:

Information overload has a significantly positive relationship with technostress.

#### H2b:

Information overload has a significantly positive relationship with exhaustion.

Life invasion (LI) is the perception that an individual is never out of reach because of technology and has to be constantly aware of work-related issues (Califf & Brooks, 2020). This will result in an undesired obscurity in the boundaries that separate their personal and work life (Zoonen et al., 2017). For instance, in today’s working world, companies have been utilizing the benefit of constant connectedness provided by social media to disseminate work-related matters to their employees (Gaudioso et al., 2017). As such, this can cause employees to feel constantly tied to their workplace even after office hours. This situation is similar in the context of mobile learning as work for students is perceived to be in terms of studying. Therefore, students may feel that the boundaries separating their personal and student life are becoming obscure because they are more accessible to their instructors via social media (Raspopovic et al., 2017). As such, they may constantly receive teaching materials from their instructors via social media platforms (Abdillah, 2016). Overall, the TS and EXH of students are posited to increase as a result of these situations. Thus, the hypotheses developed are as below:

#### H3a:

Life invasion has a significantly positive relationship with technostress.

#### H3b:

Life invasion has a significantly positive relationship with exhaustion.

Privacy invasion (PI) is defined as the feeling of worry experienced by social media users about the possible leaks of personal information on social media or the violations of their respective privacy (Gu et al., 2017). In general, social media users are becoming more worried about the security level of their personal information. This is due to the numerous data breaches that occurred recently on some of the world’s top companies (The Star, 2019). Private details which have been leaked can spread quickly and cause major problems (Kim et al., 2019). To make matters worse, numerous social media platforms today have integrated location-based services. This has left many users feeling pressured as their location becomes known to others (Zhao et al., 2012). While the capturing of location information does not directly affect RIU, it does increase TS and EXH. In many cases, as the location information gathered by social media is mainly used for marketing and advertising purposes, the increased exposure of user privacy information can seem like a stalking effect, monitoring users without their knowledge (Cooney, 2014). In addition, this has resulted in an increased worry about what these social media sites do with these details. Thus, the intrusion of one’s privacy causes a stressful effect on the psychological well-being of the users. In the mobile learning setting, there is a growing concern regarding the leak of students’ personal details contained in the submission of digital assignments, dissemination of marks awarded, and so on. This concern on privacy is posited to cause students to experience TS and EXH. As such, the hypotheses below were developed:

#### H4a:

Privacy invasion has a significantly positive relationship with technostress.

#### H4b:

Privacy invasion has a significantly positive relationship with exhaustion.

### Organism-Response Relationship

Technostress (TS) is defined as a state of stress experienced by users psychologically when engaging in technology that is superseded by physical and biological manifestations (Cao & Sun, 2018). It is the feeling of being overwhelmed or inability to deal with the mental or emotional pressure caused by the overuse of social media and mobile technologies (Ragu-Nathan et al., 2008; Zheng & Lee, 2016). Meanwhile, exhaustion (EXH) refers to a person’s aversive and inherently negative psychological reaction to stressful situations when utilizing social media (Maier et al., 2015). Overall, TS refers to the negative state in which a person develops when encountering a demanding situation related to technology while EXH refers to the individual’s feeling of tiredness as a result of the stressful situation.

In the research done by Shi et al. (2020), TS was shown to be significantly related to feelings of EXH. In addition, Maier et al. (2015) stated that TS plays a significant role in developing individuals’ intention to reduce or cease their usage. Particularly in this study, TS is an undesirable feeling encountered by students when utilizing mobile learning via social media. Thus, in order to reduce its negative impacts, students will opt to consciously control their usage intensity (Yao & Cao, 2017). One form of conscious control is the development of intention to reduce the usage as a behavioral response and adaptive strategy to these negative impacts of TS. Hence, the hypotheses below were developed:

#### H5:

Technostress has a significantly positive relationship with exhaustion.

#### H7:

Technostress has a significantly positive relationship with reduced intention to use.

Besides that, several past studies have found that EXH has a positive and significant relationship with the intention to reduce usage (Cao et al., 2020; Lin et al., 2020). In terms of mobile learning via social media, EXH will lead to the reduction of usage intention. This can be manifested in terms of taking short breaks, controlling the usage intensity, or temporarily suspending all related activities (Ravindran et al., 2014). Thus, the following hypothesis was developed:

#### H7:

Exhaustion has a significantly positive relationship with reduced intention to use.

**Fig. 1:**
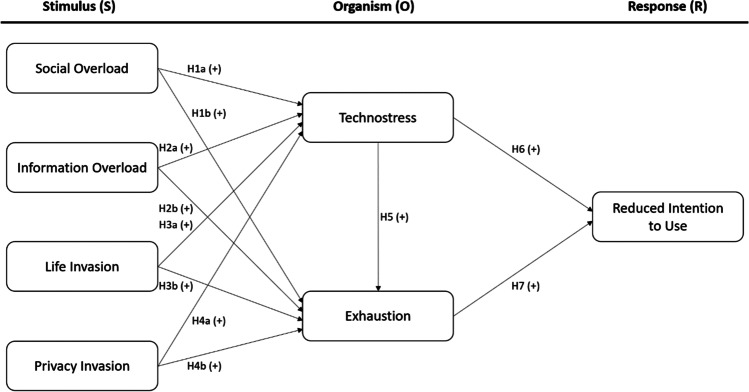
Proposed conceptual model

In view of the above-mentioned, Fig. 1 illustrates the proposed conceptual model.

## Research Methodology

As the subject matter of this study is in the area of learning, the target respondents were university students. In particular, the responses were obtained from students in Malaysian public and private universities. This is because Malaysia is renowned for its diverse multicultural society which is also reflected in the composition of university students in Malaysia (Menon & Chin, 2020). Furthermore, a substantial proportion of university students in Malaysia is made up of international students (Yunus, 2020). In addition to that, Malaysia is home to a number of branch campuses of international universities such as the University of Nottingham, Monash University, and Curtin University. Besides, many Malaysian universities have been featured on the lists of prominent university rankings by Times Higher Education and Quacquarelli Symonds. Notably, Malaysia’s higher education system was ranked 25^th^ in the Quacquarelli Symonds’ Higher Education System Strength Rankings (Quacquarelli Symonds, 2021).

As universities were unwilling to release the list of all their students, no sampling frame was available. Therefore, a non-probability sampling method in the form of purposive sampling and was utilized. This is to allow the data collected to better reflect the situation by ensuring the proper selection of participants (Yan et al., 2021). In this case, the participants have to be university students. Conversely, responses were not obtained from the universities’ alumni and staff.

As the subject matter of this study is in the online setting, an online survey in which all items were developed with reference to past studies was utilized for the data collection. Similar to other studies in the area of education during the COVID-19 pandemic (Haider & Al-Salman, 2020; Trung et al., 2020), the snowball technique was utilized to supplement the dissemination of the online survey to university students in Malaysia. More specifically, the initial contact was made with a number of university students. As an invitation to participate in the study, the link to the online survey was sent to them. These individuals were then asked to assist in identifying and approaching other university students in Malaysia to complete the study. In particular, Facebook and WhatsApp were the social media platforms selected for dissemination in view of their prevalent use (Clement, 2020). 

There are two sections to the questionnaire. In the first section, the participants were to answer questions regarding their respective demographic profiles (refer to Table 1 for the demographic profile of these respondents). Furthermore, the second section is made up of the construct items as detailed in Table 2 with the corresponding sources that were gauged with a 7-point Likert scale. Prior to the actual survey, the questionnaire was first pre-tested with three academic experts to verify the content for validity which led to a number of alterations to the questionnaire items. After that, a pilot test with 30 university students who were active on social media further guaranteed the reliability of the questionnaire items.

**Table 1:** Descriptive characteristics of the participants

Demographic characteristics	Description	Count	Percentage (%)
Gender	Male	171	44.5
	Female	213	55.5
Age	20 years and below	211	54.9
	21–25 years old	148	38.5
	26–30 years old	4	1.0
	31–35 years old	5	1.3
	36–40 years old	5	1.3
	Above 40 years old	11	2.9
Field of study	Arts and Social Science	88	22.9
	Business, Finance, and Accountancy	231	60.2
	Computer Science and IT	51	13.3
	Engineering	5	1.3
	Medicine and Health Science	8	2.1
	Physical Science	1	0.3
Number of mobile devices owned	Less than 3 devices	280	72.9
	3 to 5 devices	95	24.7
	More than 5 devices	9	2.3
SNS used for academic purposes	Facebook	249	64.8
(may choose more than one)	Instagram	180	46.9
	Line	17	4.4
	LinkedIn	24	6.3
	Pinterest	28	7.3
	Skype	17	4.4
	Snapchat	42	10.9
	Tumblr	2	0.5
	Twitter	47	12.2
	WeChat	192	50.0
	WhatsApp	348	90.6
	YouTube	255	66.4
	Others	7	1.8
Frequency of Using SNS for learning	Less than 2 h	50	13.0
(per week)	2 to less than 4 h	118	30.7
	4 to less than 6 h	79	20.6
	6 to less than 8 h	45	11.7
	8 h or more	92	24.0

**Table 2:** Constructs and adapted sources of survey items

Constructs	Measurement items	Sources
Social overload	SO1: I take too much care of my friends’ well-being on mobile social networking sites when learning	Maier et al. (2014)
	SO2: I deal too much with my friends’ problems on mobile social networking sites when learning	
	SO3: My sense of being responsible for how much fun my friends have on mobile social networking sites when learning is too strong	
	SO4: I am too caring for my friends on mobile social networking sites when learning	
	SO5: I pay too much attention to posts of my friends on mobile social networking sites during learning	
	SO6: I congratulate friends on mobile social networking sites as a consequence of the birthday reminder, although I would not congratulate them in real life	
Information overload	IO1: I am often distracted by the excessive amount of information available to me on mobile social networking sites when learning	Zhang et al. (2016)
	IO2: I find that I am overwhelmed by the amount of information I have to process on a daily basis on mobile social networking sites when learning	
	IO3: There is too much information about my friends on mobile social networking sites when learning so I find it a burden to handle	
	IO4: I find that only a small part of the information on mobile social networking sites is relevant to my learning needs	
Life invasion	LI1: Using mobile social networking sites for learning blurs the boundaries between my student and personal life	Xiao and Mou (2019)
	LI2: I have to be in touch with my studies even during my off days because of the usage of mobile social networking sites for learning	
	LI3: I have to sacrifice my weekends to keep myself updated on new mobile social networking sites for learning	
	LI4: I feel my personal life is being invaded by the usage of mobile social networking sites for learning	
Privacy invasion	PI1: I have had somebody who I do not know share my mobile social networking sites account information without authorization	Kim et al. (2019)
	PI2: I have had somebody illegally use my photo on my mobile social networking sites account	
	PI3: My learning comments and opinions on mobile social networking sites have been stolen by someone I do not know who reposted them as if the comments and opinions were theirs	
Technostress	TS1: I am forced to change my learning habits to adapt to new developments on mobile social networking sites for learning	Luqman et al. (2017)
	TS2: I have to sacrifice my personal learning time to keep up with new updates of mobile social networking sites for learning	
	TS3: I feel that my personal life is being invaded by mobile social networking sites features	
	TS4: I am threatened by people with the latest mobile social networking site skills for learning	
Exhaustion	EXH1: I feel drained from learning activities that require me to use mobile social networking sites	Cao and Sun (2018)
	EXH2: I feel tired from my usage of mobile social networking sites for learning activities	
	EXH3: Using social networking sites for learning is a strain for me	
Reduced intention to use	RIU1: I will reduce learning via mobile social networking sites	Osatuyi and Turel (2020)
	RIU2: I want to have a certain period of time during which I do not learn via mobile social networking sites	
	RIU3: I plan to stop using mobile social networking sites to learn	
	RIU4: I will not continue to use mobile social networking sites for learning	

As recommended by Hair et al. (2017), G*Power was utilized to suggest a minimum sample size. From the settings of 6 predictors and 15% effect size, 5% alpha level, and 80% power, it was calculated that the sample size for this study is 98. Overall, 384 responses were collected which is much higher than the suggested minimum sample size. Out of the 384 responses, 55.5% of them were female as shown in Table 1. In addition, they were predominantly 25 years old and below, own less than three mobile devices, as well as studying in the fields of business, finance, accountancy, arts, and social science. Moreover, the most used social media platforms for academic purposes are WeChat, YouTube, Facebook, Instagram, and WhatsApp.

## Data Analysis

### Assessing the Outer Measurement Model

Based on Table 4, all values for composite reliability and rho_A are above 0.7 which indicates that all constructs adapted into this research are reliable (Loh et al., 2019; Tamilmani et al., 2020). Moreover, convergent validity was ascertained based on the values of factor loadings and average variance extracted (AVE) (Foo et al., 2018). With regards to the same table, all outer loadings are above the threshold of 0.7 (Hew et al., 2019; Leong et al., 2020) except for SO6 (0.558), IO4 (0.585), LI2 (0.668), and TS4 (0.687). However, Tan and Ooi (2018, p. 1627) mentioned that an “outer loading between 0.4 and 0.7 can be accepted if other indicators with high loading can explain 50% of the AVE”. Therefore, all items were retained as all AVE values exceeded 0.50 (Tew et al., 2021). Moreover, this study investigated the discriminant validity (DV) by assessing the Hetero-Trait-Mono-Trait ratio of correlations (HTMT_.85_) (Henseler et al., 2015). According to Table 5, DV was established as all values are lower than 0.85. Furthermore, DV was established with the HTMT_inference_ assessment in view that all correlation values are lower than 1 as shown in Table 6.

**Table 4:** Outer loading, composite reliability, and average variance extracted

Latent constructs	Items	Outer loading	Composite reliability	rho_A	Average variance extracted
Social overload	SO1	0.854	0.908	0.886	0.625
	SO2	0.830			
	SO3	0.772			
	SO4	0.875			
	SO5	0.813			
	SO6	0.558			
Information overload	IO1	0.841	0.864	0.818	0.619
	IO2	0.851			
	IO3	0.839			
	IO4	0.585			
Life invasion	LI1	0.798	0.849	0.812	0.586
	LI2	0.668			
	LI3	0.722			
	LI4	0.859			
Privacy invasion	PI1	0.857	0.911	0.855	0.774
	PI2	0.881			
	PI3	0.901			
Technostress	TS1	0.786	0.872	0.812	0.631
	TS2	0.853			
	TS3	0.840			
	TS4	0.687			
Exhaustion	EXH1	0.886	0.924	0.879	0.803
	EXH2	0.899			
	EXH3	0.902			
Reduced intention to use	RIU1	0.855	0.871	0.817	0.629
	RIU2	0.712			
	RIU3	0.825			
	RIU4	0.773

**Table 5:** Hetero-trait–mono-trait ratio (HTMT_.85_)

Latent construct	SO	IO	LI	PI	TS	EXH	RIU
Social overload							
Information overload	0.665						
Life invasion	0.544	0.708					
Privacy invasion	0.513	0.325	0.390				
Technostress	0.563	0.748	0.799	0.429			
Exhaustion	0.417	0.680	0.748	0.263	0.829		
Reduced intention to use	0.293	0.538	0.387	0.350	0.592	0.594

**Table 6:** Hetero-trait–mono-trait inference (HTMT_inference_)

Constructs	Original sample (O)	Sample mean (M)	Bias	2.50%	97.50%
SO → IO	0.665	0.665	0.000	0.554	0.756
SO → LI	0.544	0.544	0.000	0.415	0.654
SO → PI	0.513	0.513	-0.001	0.401	0.610
SO → RIU	0.293	0.298	0.005	0.163	0.410
SO → EXH	0.417	0.416	-0.001	0.297	0.519
LI → IO	0.708	0.709	0.001	0.581	0.812
PI → IO	0.325	0.325	0.000	0.214	0.438
PI → LI	0.390	0.389	-0.002	0.274	0.502
TS → IO	0.748	0.748	0.000	0.630	0.840
TS → LI	0.799	0.800	0.001	0.702	0.880
TS → PI	0.429	0.428	-0.001	0.321	0.530
TS → RIU	0.592	0.592	0.000	0.464	0.701
TS → EXH	0.829	0.828	-0.001	0.754	0.892
TS → SO	0.563	0.563	0.000	0.448	0.668
EXH → IO	0.680	0.680	0.000	0.571	0.770
EXH → LI	0.748	0.748	0.000	0.654	0.827
EXH → PI	0.263	0.261	-0.002	0.156	0.368
EXH → RIU	0.594	0.594	0.000	0.489	0.682
RIU → IO	0.538	0.539	0.001	0.406	0.653
RIU → LI	0.387	0.397	0.010	0.279	0.485
RIU → PI	0.350	0.360	0.010	0.242	0.441

*SO* social overload, *IO* information overload, *LI* life invasion, *PI* privacy invasion, *TS* technostress, *EXH* exhaustion, *RIU* reduced intention to use

#### Statistical Analysis

The Partial Least Squares-Structural Equation Modeling (PLS-SEM) analysis for the measurement and structural model was carried out via SmartPLS. This is because Lew et al. (2020) indicated that, unlike covariance-based SEM techniques which are focused on theory testing, PLS-SEM has high predictive accuracy when it comes to complex research models with many constructs and indicators. Particularly in this case, the conceptual model includes 7 constructs and 28 indicators. Additionally, PLS-SEM is suitable for theory development as it maximizes the variance explained by the target construct (Pal et al., 2020). This is in view of the conceptualization of SO, IO, LI, PI, TS, EXH, and RIU into the SOR framework in this study. Moreover, the normality distribution of data is not required for this method of analysis. With Mardia’s multivariate, the skewness (β = 4.84, p < 0.001) and kurtosis (β = 80.96, p < 0.001) values show that the data was not normally distributed. Thus, it establishes the suitability for the utilization of PLS-SEM in this study.

##### Common Method Bias (CMB)

As only one method was used for collecting data, there may be the presence of CMB. Thus, a common method factor analysis was used to assess this bias (Lau et al., 2021). With reference to Table 3, all Ra were statistically significant at p < 0.001 with an average of 0.81. Furthermore, the average of Ra^2^ is greater than Rb^2^ which indicated that CMB is not an issue in this study.

**Table 3:** Common method factor analysis

Latent construct	Indicators	Substantive factor loading (Ra)	Ra^2^	Method factor loading (Rb)	Rb^2^
SO	SO1	0.85	0.72	0.01	0.00
	SO2	0.79	0.63	0.04	0.00
	SO3	0.90	0.81	-0.15	0.02
	SO4	0.89	0.79	-0.01	0.00
	SO5	0.81	0.65	0.01	0.00
	SO6	0.43	0.19	0.13	0.02
IO	IO1	0.95	0.90	-0.14	0.02
	IO2	0.89	0.79	-0.05	0.00
	IO3	0.71	0.51	0.16	0.02
	IO4	0.56	0.31	0.05	0.00
LI	LI1	0.56	0.32	0.26	0.07
	LI2	0.97	0.94	-0.33	0.11
	LI3	0.94	0.88	-0.24	0.06
	LI4	0.67	0.44	0.22	0.05
PI	PI1	0.84	0.70	0.05	0.00
	PI2	0.88	0.78	-0.01	0.00
	PI3	0.92	0.85	-0.04	0.00
TS	TS1	0.87	0.76	-0.11	0.01
	TS2	0.95	0.90	-0.12	0.01
	TS3	0.80	0.64	0.05	0.00
	TS4	0.53	0.28	0.20	0.04
EXH	EXH1	0.85	0.73	0.04	0.00
	EXH2	0.98	0.95	-0.09	0.01
	EXH3	0.86	0.74	0.05	0.00
RIU	RIU1	0.78	0.61	0.10	0.01
	RIU2	0.60	0.83	-0.08	0.01
	RIU3	0.91	0.77	-0.09	0.01
	RIU4	0.88	0.35	0.08	0.01
	***Average***	***0.81***	***0.67***	***0.00***	***0.02***

*SO* social overload, *IO* information overload, *LI* life invasion, *PI* privacy invasion, *TS* technostress, *EXH* exhaustion, *RIU* reduced intention to use

##### Inspecting the Inner Structural Model

**Fig. 2:**
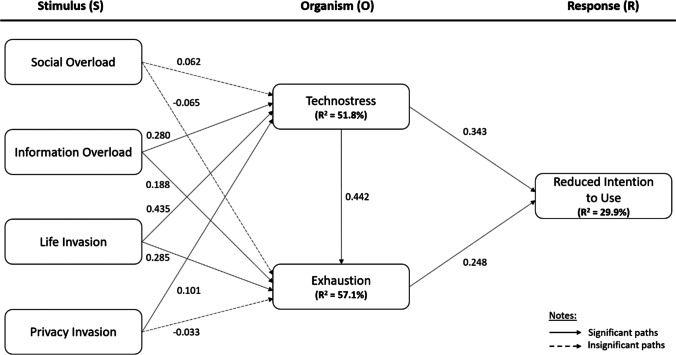
Structural model testing

Multicollinearity for all constructs was assessed using the variance inflation factor (VIF). Since all VIF values (1.289 to 2.074) are below 5, this indicates that multicollinearity is not a problem in this study (Loh et al., 2020). With reference to Fig. 2 and Table 7, all hypotheses were supported except H1a, H1b, and H4b. In other words, TS is significantly influenced by IO (β = 0.280, p < 0.001), LI (β = 0.435, p < 0.001), and PI (β = 0.101, p < 0.05) whereas EXH is significantly influenced by IO (β = 0.188, p < 0.001), LI (β = 0.285, p < 0.001), and TS (β = 0.442, p < 0.001). Subsequently, TS (β = 0.248, p < 0.01) as well as EXH (β = 0.343, p < 0.001) are significant antecedents of RIU. Table 8 indicates that the model has low predictive power since the majority of indicators under PLS produced higher root mean squared errors (RMSE) compared to the linear regression model (LM) (Shmueli et al., 2019).

**Table 7:** Hypotheses testing

Hypotheses	PLS Paths	Original sample (O)	Sample mean (M)	Standard deviation (STDEV)	T statistics (|O/STDEV|)	p-values	Bias corrected confidence interval	Remarks
H1a	SO → TS^NS^	0.062	0.064	0.061	1.018	0.309	[-0.054, 0.185]	Not Significant
H1b	SO → EXH^NS^	-0.065	-0.066	0.049	1.315	0.189	[-0.161, 0.034]	Not Significant
H2a	IO → TS***	0.280	0.279	0.064	4.348	0.000	[0.159, 0.410]	Significant
H2b	IO → EXH***	0.188	0.192	0.056	3.340	0.000	[0.085, 0.303]	Significant
H3a	LI → TS***	0.435	0.436	0.054	8.021	0.000	[0.324, 0.534]	Significant
H3b	LI → EXH***	0.285	0.284	0.057	4.960	0.000	[0.175, 0.402]	Significant
H4a	PI → TS*	0.101	0.100	0.044	2.313	0.021	[0.015, 0.185]	Significant
H4b	PI → EXH^NS^	-0.033	-0.032	0.038	0.867	0.386	[-0.105, 0.043]	Not Significant
H5	TS → EXH***	0.442	0.440	0.059	7.491	0.000	[0.324, 0.555]	Significant
H6	TS → RIU**	0.248	0.251	0.074	3.351	0.001	[0.097, 0.388]	Significant
H7	EXH → RIU***	0.343	0.344	0.067	5.092	0.000	[0.208, 0.473]	Significant

*RIU* reduced intention to use, *SO* social overload, *IO* information overload, *LI* life invasion, *PI* privacy invasion, *TS* technostress, *EXH* exhaustion

^*^p < 0.05; **p < 0.01; ***p < 0.001; ^NS^ Not supported

**Table 8:** PLSpredict

	PLS			LM	
Reduced intention to use	Q^2^_predict	RMSE	MAE	RMSE	MAE
RIU1	0.154	1.443	1.150	1.431	1.113
RIU2	0.072	1.659	1.369	1.614	1.305
RIU3	0.064	1.705	1.412	1.606	1.293
RIU4	0.123	1.588	1.260	1.590	1.246

##### Predictive Relevance, Predictive Power, and Effect Size

With reference to Tables 8 and 9, the research model was discovered to possess predictive relevance as all values of Q^2^ are more than 0 (Lee et al., 2020; Wong et al., 2015). In addition to that, the model was able to capture 51.8%, 57.1%, and 29.9% of the variances in TS, EXH, and RIU respectively. The effect size which indicates the intensities of associations between variables is considered to be small, medium, or large based on the f^2^ value thresholds of 0.02, 0.15, and 0.35 respectively (Wong et al., 2016; Yan et al., 2021). Moreover, there is no effect if the f^2^ has a value of less than 0.02 (Lew et al., 2020). Based on Table 10, there are small effects for IO and LI with EXH, IO with TS, as well as TS and EXH with RIU. Besides, medium effects are present between LI with TS as well as TS with EXH whereas SO and PI do not affect both TS and EXH.

**Table 9:** Predictive relevance (Q^2^) and predictive power (R^2^)

Constructs	SSO	SSE	Q^2^ (= 1-SSE/SSO)	R^2^
Social overload	2304.000	2304.000		
Information overload	1536.000	1536.000		
Life invasion	1536.000	1536.000		
Privacy invasion	1152.000	1152.000		
Technostress	1536.000	1044.562	0.320	0.518
Exhaustion	1152.000	639.888	0.445	0.571
Reduced intention to use	1536.000	1270.019	0.173	0.299

**Table 10:** Effect size (f^2^)

Predictor constructs / dependent constructs	SO	IO	LI	PI	TS	EXH	RIU
Social overload					0.005	0.006	
Information overload					0.090	0.042	
Life invasion					0.240	0.093	
Privacy invasion					0.017	0.002	
Technostress						0.220	0.045
Exhaustion							0.085
Reduced intention to use							

##### Importance Performance Map Analysis (IPMA)

**Fig. 3:**
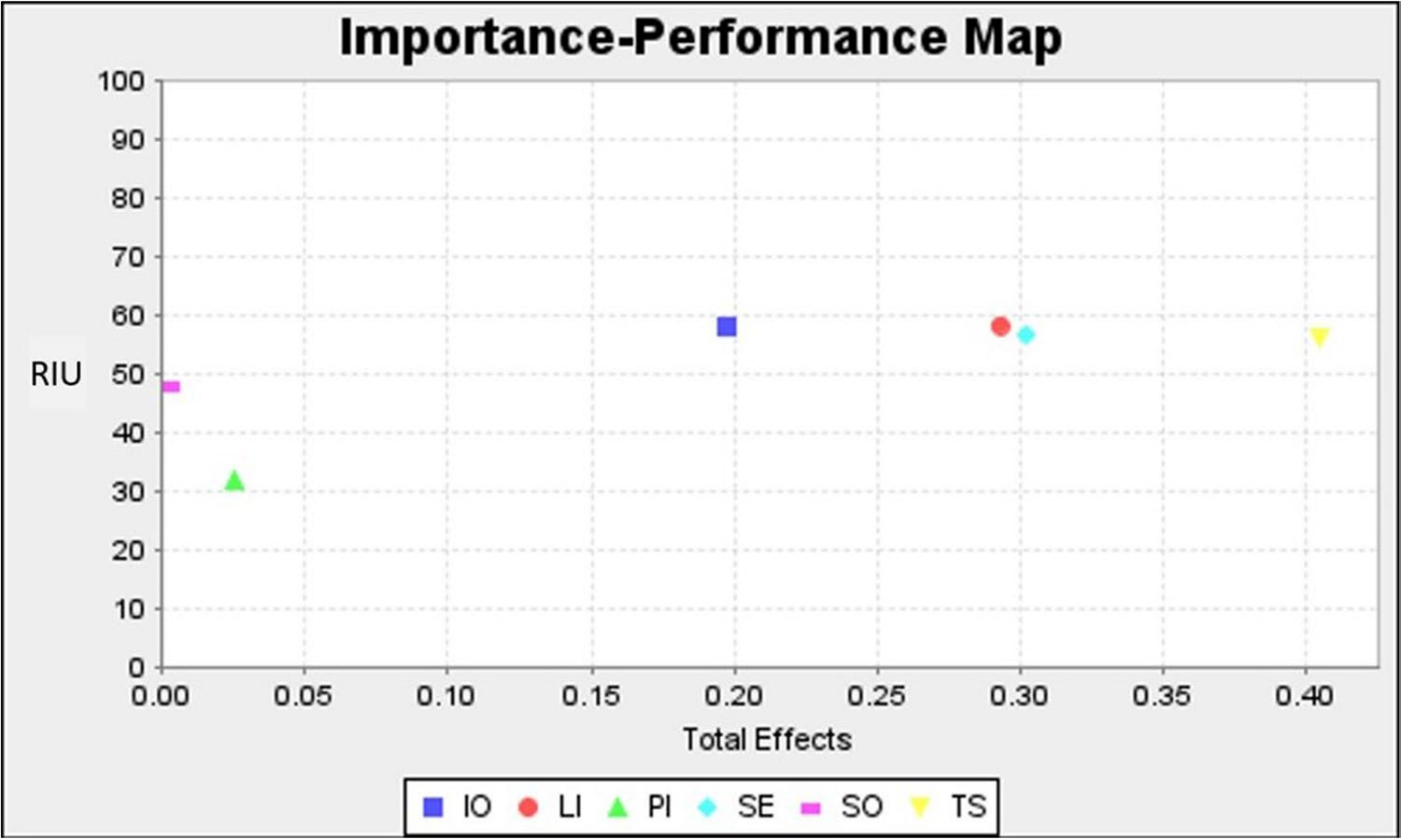
Importance performance map analysis

Table 11 and Fig. 3 show the results of the IPMA. According to Higueras-Castillo et al., (2019, p. 393) “IMPA allows researchers to identify the importance (based on the total effects) and performance (based on the average latent variable score from 0 to 100) of the exogenous constructs for a specific endogenous construct, which could help in highlighting the specific focus within a complex research model”. It was revealed that nearly all of the variables’ performances are within the range of 45 to 60. Furthermore, TS should be given priority as it was found to have high importance (0.404) but low performance (56.078).

**Table 11:** Importance performance map results

Latent variables	Importance (Total Effect)	Performance (Index value)
Social overload	0.003	47.658
Information overload	0.197	58.139
Life invasion	0.293	58.284
Privacy invasion	0.025	32.000
Technostress	0.404	56.078
Exhaustion	0.302	56.776
Mean value	0.200	51.490

## Discussion

This research was conducted due to the rising importance of mobile learning via social media. Responses were collected from university students to which the demographics were found to closely match official statistics. More specifically, there are more female than male students, mostly 25 years old and below, as well as pursuing arts and business programmes (Ministry of Higher Education Malaysia, 2020). Empirically, H1a and H1b were unsupported which indicates that SO does not have significantly positive relationships with TS and EXH respectively. This is because even under ordinary circumstances, the use of social media is connected with the development of an individual’s social capital and purposes. Additionally, university students belong to Generation Z which are social media natives, and view social media as a key avenue to connect with their friends (Viens, 2019). On top of that, the process of learning generally takes place in a collectivistic setting. Therefore, SO is not significant in this context as the social aspect present in a physical classroom was merely translated to the virtual one. In contrast, IO was discovered to possess significantly positive relationships with both TS and EXH, thereby supporting H2a and H2b respectively. In this study, mobile social media platforms are used for the dissemination of information which is part and parcel of the teaching and learning process. As a result, on top of the information obtained from existing formal channels such as the university’s own e-learning platform, students are also exposed to additional information that is related to their studies on mobile social media platforms.

Moreover, the support for H3a and H3b indicates that LI has significantly positive relationships with both TS and EXH respectively. This is attributed to the invasive nature of mobile social media that distorts the boundaries of personal and work life. More specifically, mobile learning via social media enables students to constantly be connected with their instructors. This is in addition to the fact that social media tends to be perceived as a platform for entertainment instead of being used for work; which to students, is in the form of studying (Maguire, 2020). Thus, students find it hard to balance between their personal and study lives which causes them to feel exhausted and stressed. Besides, as PI has a significant relationship with TS but not with EXH, H4a is supported while H4b is not. In other words, PI does not lead to EXH as students can take simple remedial measures such as adjusting their privacy settings. However, students will experience TS due to the lingering privacy concerns from the content they create for mobile learning via social media despite the remedial measures. This is because the online content such as video assignments would include the students’ personal details that can be shared by their friends to a wide audience who may be strangers to them.

Furthermore, H5 is supported given that there is a significantly positive association between TS and EXH. Additionally, TS and EXH are significantly positive antecedents of RIU which provides empirical support for H6 and H7 respectively. Overall, the stimuli from using mobile learning via social media will cause students to experience TS and EXH. In particular, students will face stressful situations when using mobile learning via social media which will then cause them to feel fatigued. As such, the experience of both states will drive students to develop behavioral responses to cope with these situations by reducing their usage of mobile learning via social media.

### Implications

Practically, as mobile learning via social media is becoming more prevalent due to the COVID-19 pandemic, the findings will help educators to improve on their teaching methods to better shape the learning experiences of their students. Given the significance of IO, LI, and EXH, educators should look to regulate the dissemination of information. This can be in terms of timing throughout the day and amount within a certain period of time. Educators should identify the most optimal time to share these contents and schedule them accordingly by using tools such as AgoraPulse. These practices will also help to ensure that students are more engaged when these contents are shared.

Moreover, social media companies also play an important role in this situation given that the technical aspects of mobile social media contribute to the reduced intention to use it for learning. Therefore, social media companies should look to improve features that would make it easier for students to use mobile learning via social media. Since mobile social media is segregated from the universities’ official e-learning platforms, the difficulty in sharing files between platforms can cause TS. In view of this, social media companies should collaborate with universities to enable the seamless sharing of documents between platforms.

Theoretically, this study further developed on the comprehension of online teaching and learning from past literature (e.g., Raspopovic & Jankulovic, 2017; Teo et al., 2020). More specifically, the present study adds to the understanding of the reduced intention to use mobile learning via social media. Using a uniquely developed SOR framework, the research model looks into the effects of various antecedents on the reduced intention to use mobile learning. In particular, this study examined TS more comprehensively by including several technostress-creators into the SOR model. Hence, this study’s research model has shown that technostress-creators can be feasibly integrated as a theoretical approach to examining these variables. Furthermore, the comprehension of this subject matter was contextualized to the COVID-19 setting which not many studies have yet to do given the recency of the pandemic. Moreover, the relevance of the COVID-19 pandemic on the subject matter further highlights this study's theoretical implications of this study.

### Limitations and Future Directions

Firstly, this research was only conducted in Malaysia. Therefore, the insights of this study may not be wholly generalizable to reflect the online and mobile learning landscape in other countries. This is due to the difference in the forms of culture, pedagogy, and others among different countries. These variations would undeniably influence the experience of students’ utilization of mobile learning via social media in other countries. Hence, future studies should consider conducting a study in which the data collection is carried out in several countries. Additionally, this research was conducted using a cross-sectional approach. As such, whether the effects of TS and EXH will increase or decrease after students use mobile learning via social media over an extended period of time is unaddressed. Therefore, future studies can consider replicating this study into a longitudinal one. This will allow for the comparison of variances over different periods which would lead to a more comprehensive study. Lastly, this study developed the research model according to the SOR framework. With that said, the factors that represented the stimulus (i.e., SO, IO, LI, and PI) were not hypothesized to have any direct effect on the response (i.e., RIU). Therefore, future studies can look into making alterations to this study’s research model in addition to the inclusion of moderators and mediators  to address the above-mentioned limitation.

## Conclusion

The COVID-19 pandemic has brought about numerous ramifications to many sectors around the world, including higher education (Choudrie et al., 2021). The abrupt shift from physical to online classes is one of the most significant effects of the pandemic to the higher education sector (Dwivedi et al., 2020; Iivari et al., 2020; Verma & Gustafsson, 2020). As a response, this study developed and utilized a novel theoretical model to look into online learning, with the focus on mobile social media. More specifically, technostress and exhaustion were discovered to be significant antecedents of reduced intention to use mobile learning via social media. This should be a huge concern for all stakeholders in the higher education sector as students are already facing many other issues due to the COVID-19 pandemic. In addition, this study shows that the characteristics inherent to mobile learning can result in detrimental impacts (i.e., TS and EXH) on university students. Ultimately, depending on the level of reduced intention, it can even lead to discontinuance intention. In this case, it is manifested by the situation in which university students consider to or actually drop out of university (Rimmer et al., 2021). With that said, several measures were proposed by this study to help provide relief to the situation. As the COVID-19 pandemic will linger on as an issue in the foreseeable future, stakeholders in the higher education sector will continue to encounter complications arising from this situation. Thus, research in the area of mobile learning and its variants should be further enhanced given its significance to the current and future situations.
